# Supplementary material for: Emergence of phenotypic and genotypic antimicrobial resistance in Mycobacterium tuberculosis
Source: Sci Rep. 2022 Dec 11;12:21429. doi: 10.1038/s41598-022-25827-6 (PMC9742156; doi:10.1038/s41598-022-25827-6)
Supplement: Supplementary file 2 — Supplementary Information 2. [file 41598_2022_25827_MOESM2_ESM.docx]

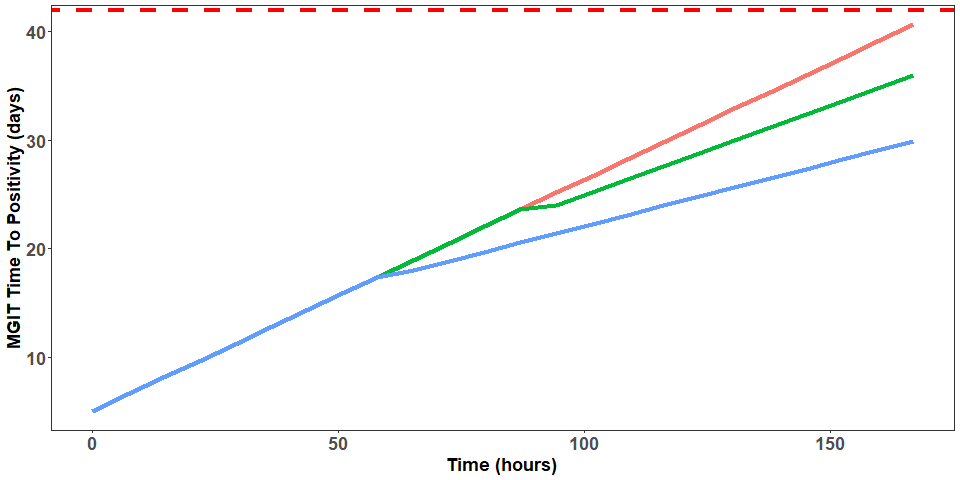


Fig. S1. Visual representation of a mono- (red) and bi-phasic (blue and green) MGIT TTP-time kill series with the horizonal red dashed line representing the upper limit of quantification (i.e. 42 days).


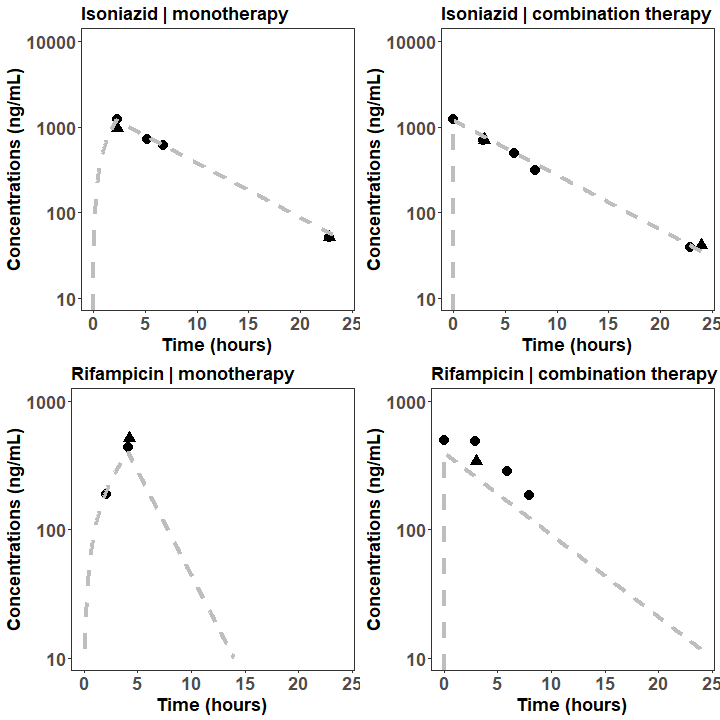


Fig. S2. Simulated (grey dashed line) versus observed pharmacokinetic profile in central reservoir (circles) and extra capillary space (triangles).


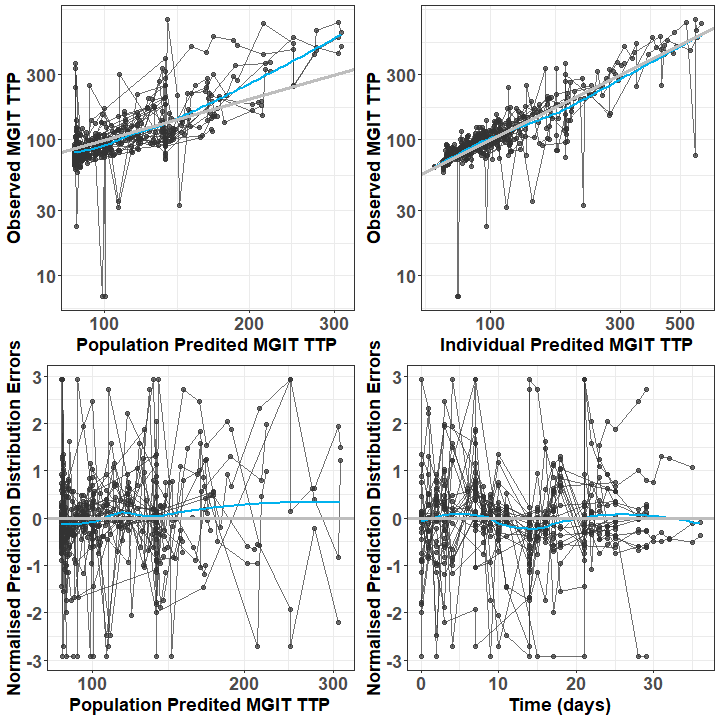


Fig. S3. Basic Goodness of Fitness plots Pharmacodynamic model. Grey and blue lines represent the line of identity and local polynomial regression fitting, respectively.

Table S1. Summary of WGS data.

| **id** | **project_title** | **scientific_name** | **strain** | **sample_alias** | **sample_description** |
| --- | --- | --- | --- | --- | --- |
| ERS12274705 | PRJEB53040 | Mycobacterium tuberculosis | H37Rv | E1_0-25R-0-25I-2 | 1/4 MIC RIF and 1/4 MIC INH, end of evolution cycle 2, replicate 1 |
| ERS12274706 | PRJEB53040 | Mycobacterium tuberculosis | H37Rv | E1_0-25R-0I-2 | 1/4 MIC RIF and 0 MIC INH, end of evolution cycle 2, replicate 1 |
| ERS12274707 | PRJEB53040 | Mycobacterium tuberculosis | H37Rv | E1_0R-0-25I-2 | 0 MIC RIF and 1/4 MIC INH, end of evolution cycle 2, replicate 1 |
| ERS12274708 | PRJEB53040 | Mycobacterium tuberculosis | H37Rv | E1_0R-0I-2 | 0 MIC RIF and 0 MIC INH, end of evolution cycle 2, replicate 1 |
| ERS12274709 | PRJEB53040 | Mycobacterium tuberculosis | H37Rv | E1_0R-1I-2 | 0 MIC RIF and 1 MIC INH, end of evolution cycle 2, replicate 1 |
| ERS12274710 | PRJEB53040 | Mycobacterium tuberculosis | H37Rv | E1_0R-8I-2 | 0 MIC RIF and 8 MIC INH, end of evolution cycle 2, replicate 1 |
| ERS12274711 | PRJEB53040 | Mycobacterium tuberculosis | H37Rv | E1_1R-0I-2 | 1 MIC RIF and 0 MIC INH, end of evolution cycle 2, replicate 1 |
| ERS12274712 | PRJEB53040 | Mycobacterium tuberculosis | H37Rv | E1_1R-1I-2 | 1 MIC RIF and 1 MIC INH, end of evolution cycle 2, replicate 1 |
| ERS12274713 | PRJEB53040 | Mycobacterium tuberculosis | H37Rv | E1_8R-0I-2 | 8 MIC RIF and 0 MIC INH, end of evolution cycle 2, replicate 1 |
| ERS12274714 | PRJEB53040 | Mycobacterium tuberculosis | H37Rv | E1_8R-8I-2 | 8 MIC RIF and 8 MIC INH, end of evolution cycle 2, replicate 1 |
| ERS12274715 | PRJEB53040 | Mycobacterium tuberculosis | H37Rv | E1_Start-culture-2 | Start culture, replicate 1 |
| ERS12274716 | PRJEB53040 | Mycobacterium tuberculosis | H37Rv | E2_0-25R-0-25I-2 | 1/4 MIC RIF and 1/4 MIC INH, end of evolution cycle 2, replicate 2 |
| ERS12274717 | PRJEB53040 | Mycobacterium tuberculosis | H37Rv | E2_0-25R-0I-2 | 1/4 MIC RIF and 0 MIC INH, end of evolution cycle 2, replicate 2 |
| ERS12274718 | PRJEB53040 | Mycobacterium tuberculosis | H37Rv | E2_0R-0-25I-2 | 0 MIC RIF and 1/4 MIC INH, end of evolution cycle 2, replicate 2 |
| ERS12274719 | PRJEB53040 | Mycobacterium tuberculosis | H37Rv | E2_0R-0I-2 | 0 MIC RIF and 0 MIC INH, end of evolution cycle 2, replicate 2 |
| ERS12274720 | PRJEB53040 | Mycobacterium tuberculosis | H37Rv | E2_0R-1I-2 | 0 MIC RIF and 1 MIC INH, end of evolution cycle 2, replicate 2 |
| ERS12274721 | PRJEB53040 | Mycobacterium tuberculosis | H37Rv | E2_0R-8I-2 | 0 MIC RIF and 8 MIC INH, end of evolution cycle 2, replicate 2 |
| ERS12274722 | PRJEB53040 | Mycobacterium tuberculosis | H37Rv | E2_1R-0I-2 | 1 MIC RIF and 0 MIC INH, end of evolution cycle 2, replicate 2 |
| ERS12274723 | PRJEB53040 | Mycobacterium tuberculosis | H37Rv | E2_1R-1I-2 | 1 MIC RIF and 1 MIC INH, end of evolution cycle 2, replicate 2 |
| ERS12274724 | PRJEB53040 | Mycobacterium tuberculosis | H37Rv | E2_8R-0I-2 | 8 MIC RIF and 0 MIC INH, end of evolution cycle 2, replicate 2 |
| ERS12274725 | PRJEB53040 | Mycobacterium tuberculosis | H37Rv | E2_8R-8I-2 | 8 MIC RIF and 8 MIC INH, end of evolution cycle 2, replicate 2 |
| ERS12274726 | PRJEB53040 | Mycobacterium tuberculosis | H37Rv | E2_Start-culture-2 | Start culture, replicate 2 |
| ERS12274727 | PRJEB53040 | Mycobacterium tuberculosis | H37Rv | 0R-0I-7 | 0 MIC RIF and 0 MIC INH, end of evolution cycle 1, replicate 2 |
| ERS12274728 | PRJEB53040 | Mycobacterium tuberculosis | H37Rv | 0R-1-4I-1 | 0 MIC RIF and 1/4 MIC INH, end of evolution cycle 1, replicate 2 |
| ERS12274729 | PRJEB53040 | Mycobacterium tuberculosis | H37Rv | 0R-1-4I-9 | 0 MIC RIF and 1/4 MIC INH, end of evolution cycle 1, replicate 1 |
| ERS12274730 | PRJEB53040 | Mycobacterium tuberculosis | H37Rv | 0R-1I-5 | 0 MIC RIF and 1 MIC INH, end of evolution cycle 1, replicate 2 |
| ERS12274731 | PRJEB53040 | Mycobacterium tuberculosis | H37Rv | 0R-8I-8 | 0 MIC RIF and 8 MIC INH, end of evolution cycle 1, replicate 1 |
| ERS12274732 | PRJEB53040 | Mycobacterium tuberculosis | H37Rv | 0R-8I-11 | 0 MIC RIF and 8 MIC INH, end of evolution cycle 1, replicate 2 |
| ERS12274733 | PRJEB53040 | Mycobacterium tuberculosis | H37Rv | 1-4R-1-4I-2 | 1/4 MIC RIF and 1/4 MIC INH, end of evolution cycle 1, replicate 2 |
| ERS12274734 | PRJEB53040 | Mycobacterium tuberculosis | H37Rv | 1-4R-1-4I-10 | 1/4 MIC RIF and 1/4 MIC INH, end of evolution cycle 1, replicate 1 |
| ERS12274735 | PRJEB53040 | Mycobacterium tuberculosis | H37Rv | 1R-1I-6 | 1 MIC RIF and 1 MIC INH, end of evolution cycle 1, replicate 2 |
| ERS12274736 | PRJEB53040 | Mycobacterium tuberculosis | H37Rv | 8R-0I-3 | 8 MIC RIF and 0 MIC INH, end of evolution cycle 1, replicate 2 |
| ERS12274737 | PRJEB53040 | Mycobacterium tuberculosis | H37Rv | 8R-8I-4 | 8 MIC RIF and 8 MIC INH, end of evolution cycle 1, replicate 2 |
| ERS12274738 | PRJEB53040 | Mycobacterium tuberculosis | H37Rv | 0R-0I-15 | 0 MIC RIF and 0 MIC INH, end of evolution cycle 1, replicate 1 |
| ERS12274739 | PRJEB53040 | Mycobacterium tuberculosis | H37Rv | 0R-1I-13 | 0 MIC RIF and 1 MIC INH, end of evolution cycle 1, replicate 1 |
| ERS12274740 | PRJEB53040 | Mycobacterium tuberculosis | H37Rv | 1-4R-0I-14 | 1/4 MIC RIF and 0 MIC INH, end of evolution cycle 1, replicate 1 |
| ERS12274741 | PRJEB53040 | Mycobacterium tuberculosis | H37Rv | 1R-0I-16 | 1 MIC RIF and 0 MIC INH, end of evolution cycle 1, replicate 2 |
| ERS12274742 | PRJEB53040 | Mycobacterium tuberculosis | H37Rv | 8R-0I-12 | 8 MIC RIF and 0 MIC INH, end of evolution cycle 1, replicate 1 |
| ERS12274743 | PRJEB53040 | Mycobacterium tuberculosis | H37Rv | Day0-Exp1 | Start 1/3 x standard dose TDS HFIM experiment |
| ERS12274744 | PRJEB53040 | Mycobacterium tuberculosis | H37Rv | Day0-Exp2 | Start 3 x standard dose OD HFIM experiment |
| ERS12274745 | PRJEB53040 | Mycobacterium tuberculosis | H37Rv | Day6-Exp1 | End 1/3 x standard dose TDS HFIM experiment |
| ERS12274746 | PRJEB53040 | Mycobacterium tuberculosis | H37Rv | Day7-Exp2 | End 3 x standard dose OD HFIM experiment |
| ERS12274747 | PRJEB53040 | Mycobacterium tuberculosis | H37Rv | H37Rv-1 | 1/4 MIC RIF and 0 MIC INH, end of evolution cycle 1, replicate 2 |
| ERS12274748 | PRJEB53040 | Mycobacterium tuberculosis | H37Rv | H37Rv-2 | 1 MIC RIF and 0 MIC INH, end of evolution cycle 1, replicate 1 |
| ERS12274749 | PRJEB53040 | Mycobacterium tuberculosis | H37Rv | H37Rv-3 | 8 MIC RIF and 8 MIC INH, end of evolution cycle 1, replicate 1 |
| ERS12274750 | PRJEB53040 | Mycobacterium tuberculosis | H37Rv | H37Rv-4 | 1 MIC RIF and 1 MIC INH, end of evolution cycle 1, replicate 1 |
| ERS12274751 | PRJEB53040 | Mycobacterium tuberculosis | H37Rv | Frank_HFA_OD_23AUG2019_end-355082158 | End standard dose OD HFIM experiment |
| ERS12274752 | PRJEB53040 | Mycobacterium tuberculosis | H37Rv | Frank_HFB_TDS_28NOV2019_start-355004492 | Start standard dose TDS HFIM experiment |
| ERS12274753 | PRJEB53040 | Mycobacterium tuberculosis | H37Rv | Frank_HFC_TDS_10DEC2019_end-355119001 | End standard dose TDS HFIM experiment |
